# The *CCP*4 suite: integrative software for macromolecular crystallography

**DOI:** 10.1107/S2059798323003595

**Published:** 2023-05-30

**Authors:** Jon Agirre, Mihaela Atanasova, Haroldas Bagdonas, Charles B. Ballard, Arnaud Baslé, James Beilsten-Edmands, Rafael J. Borges, David G. Brown, J. Javier Burgos-Mármol, John M. Berrisford, Paul S. Bond, Iracema Caballero, Lucrezia Catapano, Grzegorz Chojnowski, Atlanta G. Cook, Kevin D. Cowtan, Tristan I. Croll, Judit É. Debreczeni, Nicholas E. Devenish, Eleanor J. Dodson, Tarik R. Drevon, Paul Emsley, Gwyndaf Evans, Phil R. Evans, Maria Fando, James Foadi, Luis Fuentes-Montero, Elspeth F. Garman, Markus Gerstel, Richard J. Gildea, Kaushik Hatti, Maarten L. Hekkelman, Philipp Heuser, Soon Wen Hoh, Michael A. Hough, Huw T. Jenkins, Elisabet Jiménez, Robbie P. Joosten, Ronan M. Keegan, Nicholas Keep, Eugene B. Krissinel, Petr Kolenko, Oleg Kovalevskiy, Victor S. Lamzin, David M. Lawson, Andrey A. Lebedev, Andrew G. W. Leslie, Bernhard Lohkamp, Fei Long, Martin Malý, Airlie J. McCoy, Stuart J. McNicholas, Ana Medina, Claudia Millán, James W. Murray, Garib N. Murshudov, Robert A. Nicholls, Martin E. M. Noble, Robert Oeffner, Navraj S. Pannu, James M. Parkhurst, Nicholas Pearce, Joana Pereira, Anastassis Perrakis, Harold R. Powell, Randy J. Read, Daniel J. Rigden, William Rochira, Massimo Sammito, Filomeno Sánchez Rodríguez, George M. Sheldrick, Kathryn L. Shelley, Felix Simkovic, Adam J. Simpkin, Pavol Skubak, Egor Sobolev, Roberto A. Steiner, Kyle Stevenson, Ivo Tews, Jens M. H. Thomas, Andrea Thorn, Josep Triviño Valls, Ville Uski, Isabel Usón, Alexei Vagin, Sameer Velankar, Melanie Vollmar, Helen Walden, David Waterman, Keith S. Wilson, Martyn D. Winn, Graeme Winter, Marcin Wojdyr, Keitaro Yamashita

**Affiliations:** a York Structural Biology Laboratory, Department of Chemistry, University of York, York YO10 5DD, United Kingdom; b STFC, Rutherford Appleton Laboratory, Didcot OX11 0FA, United Kingdom; c CCP4, Research Complex at Harwell, Rutherford Appleton Laboratory, Didcot OX11 0FA, United Kingdom; d Biosciences Institute, Newcastle University, Newcastle upon Tyne NE2 4HH, United Kingdom; e Diamond Light Source, Harwell Science and Innovation Campus, Didcot OX11 0DE, United Kingdom; f The Center of Medicinal Chemistry (CQMED), Center for Molecular Biology and Genetic Engineering (CBMEG), University of Campinas (UNICAMP), Av. Dr. André Tosello 550, 13083-886 Campinas, Brazil; g Laboratoires Servier SAS Institut de Recherches, Croissy-sur-Seine, France; h Institute of Systems, Molecular and Integrative Biology, University of Liverpool, Liverpool L69 7ZB, United Kingdom; i Protein Data Bank in Europe, European Molecular Biology Laboratory, European Bioinformatics Institute (EMBL–EBI), Wellcome Genome Campus, Hinxton, Cambridge CB10 1SD, United Kingdom; j Crystallographic Methods, Institute of Molecular Biology of Barcelona (IBMB–CSIC), Barcelona Science Park, Helix Building, Baldiri Reixac 15, 08028 Barcelona, Spain; k MRC Laboratory of Molecular Biology, Francis Crick Avenue, Cambridge CB2 0QH, United Kingdom; l Randall Centre for Cell and Molecular Biophysics, Faculty of Life Sciences and Medicine, King’s College London, London SE1 9RT, United Kingdom; m European Molecular Biology Laboratory, Hamburg Unit, Notkestrasse 85, 22607 Hamburg, Germany; n The Wellcome Centre for Cell Biology, University of Edinburgh, Michael Swann Building, Max Born Crescent, The King’s Buildings, Edinburgh EH9 3BF, United Kingdom; o Department of Haematology, Cambridge Institute for Medical Research, University of Cambridge, Hills Road, Cambridge CB2 0XY, United Kingdom; p Altos Labs, Portway Building, Granta Park, Great Abington, Cambridge CB21 6GP, United Kingdom; q Discovery Sciences, R&D BioPharmaceuticals, AstraZeneca, Darwin Building, Cambridge Science Park, Milton Road, Cambridge CB4 0WG, United Kingdom; r Rosalind Franklin Institute, Harwell Science and Innovation Campus, Didcot OX11 0QS, United Kingdom; s Department of Mathematical Sciences, University of Bath, Bath, United Kingdom; t Department of Biochemistry, University of Oxford, Dorothy Crowfoot Hodgkin Building, Oxford OX1 3QU, United Kingdom; u Oncode Institute and Department of Biochemistry, Netherlands Cancer Institute, Amsterdam, The Netherlands; v European Molecular Biology Laboratory, c/o DESY, Notkestrasse 85, 22607 Hamburg, Germany; w School of Life Sciences, University of Essex, Wivenhoe Park, Colchester CO4 3SQ, United Kingdom; x Department of Biological Sciences, Institute of Structural and Molecular Biology, Birkbeck College, London WC1E 7HX, United Kingdom; y Faculty of Nuclear Sciences and Physical Engineering, Czech Technical University in Prague, Břehová 7, 115 19 Prague 1, Czech Republic; z Institute of Biotechnology of the Czech Academy of Sciences, BIOCEV, Průmyslová 55, 252 50 Vestec, Czech Republic; aa Department of Biochemistry and Metabolism, John Innes Centre, Norwich NR4 7UH, United Kingdom; bb Department of Medical Biochemistry and Biophysics, Karolinska Institutet, SE-171 77 Stockholm, Sweden; cc Biological Sciences, Institute for Life Sciences, University of Southampton, Southampton SO17 1BJ, United Kingdom; dd Department of Life Sciences, Imperial College London, South Kensington Campus, London SW7 2AZ, United Kingdom; ee Translational and Clinical Research Institute, Newcastle University, Paul O’Gorman Building, Medical School, Framlington Place, Newcastle upon Tyne NE2 4HH, United Kingdom; ff Department of Infectious Diseases, Leiden University Medical Center, PO Box 9600, 2300 RC Leiden, The Netherlands; gg Department of Physics, Chemistry and Biology (IFM), Linköping University, SE-581 83 Linköping, Sweden; hh Biozentrum and SIB Swiss Institute of Bioinformatics, University of Basel, 4056 Basel, Switzerland; ii Discovery Centre, Biologics Engineering, AstraZeneca, Biomedical Campus, 1 Francis Crick Avenue, Trumpington, Cambridge CB2 0AA, United Kingdom; jj Department of Structural Chemistry, Georg-August-Universität Göttingen, Tammannstrasse 4, 37077 Göttingen, Germany; kk Institute for Protein Design, University of Washington, Seattle, WA 98195, USA; ll Department of Biomedical Sciences, University of Padova, Italy; mm Institute for Nanostructure and Solid State Physics, Universität Hamburg, 22761 Hamburg, Germany; nn ICREA, Institució Catalana de Recerca i Estudis Avançats, Passeig Lluís Companys 23, 08003 Barcelona, Spain; oo School of Molecular Biosciences, College of Medical Veterinary and Life Sciences, University of Glasgow, Glasgow, United Kingdom; pp Scientific Computing Department, Science and Technology Facilities Council, Didcot OX11 0FA, United Kingdom; qq Global Phasing Limited (United Kingdom), Sheraton House, Castle Park, Cambridge CB3 0AX, United Kingdom; Institute of Integrative Biology, University of Liverpool, United Kingdom

**Keywords:** Collaborative Computational Project No. 4, *CCP*4, crystallography software, macromolecular crystallography

## Abstract

The Collaborative Computational Project No. 4 (CCP4) is a UK-led international collective with a mission to develop, test, distribute and promote software for macromolecular crystallography. The *CCP*4 suite is a multiplatform collection of programs brought together by familiar execution routines, a set of common libraries and graphical interfaces. The *CCP*4 suite has experienced several considerable changes since its last reference article, involving new infrastructure, original programs and graphical interfaces. This article, which is intended as a general literature citation for the use of the *CCP*4 software suite in structure determination, will guide the reader through such transformations, offering a general overview of the new features and outlining future developments. As such, it aims to highlight the individual programs that comprise the suite and to provide the latest references to them for perusal by crystallographers around the world.

## Abstract 2

This article describes the *Collaborative Computational Project No.* 4 (*CCP*4). It is intended as a general literature citation for the use of the *CCP*4 software suite in structure determination.

## 1. Introduction

**Figure 1:**
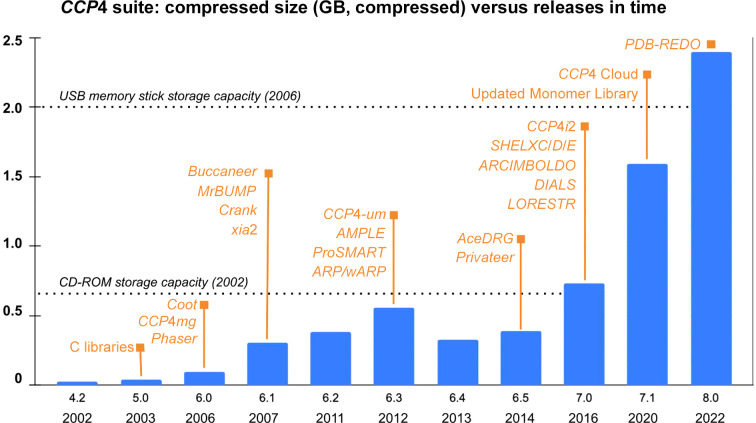
Evolution in the size of the *CCP*4 suite from version 4.2 (2002) through to version 8.0 (2022). Some representative programs included in the releases are highlighted in orange. The update mechanism (*CCP*4-*um*) was first used in version 6.3. New graphical interfaces were introduced in versions 7.0 (*CCP*4*i*2) and 7.1 (*CCP*4 Cloud). *Coot* and *CCP*4*mg* were originally distributed separately, but were bundled with the suite from version 6.5. For reference, the sizes of two popular contemporary storage devices are shown as dotted lines; please note that these were never targeted as distribution media.

As a technique, macromolecular crystallography (MX) relies heavily on computational methods, built on top of a strict set of conventions and common formats. Most conventions follow the lead of the International Union of Crystallography (IUCr), while MX software development is undertaken by both academic and private sector initiatives, such as the Phenix Consortium (Liebschner *et al.*, 2019 ▸) and Global Phasing Ltd (Cambridge, United Kingdom). Based in the UK, MX software tools find a common distribution and maintenance channel under the umbrella of the Collaborative Computational Project No. 4, best known as CCP4. This consortium was established by the UK Science Research Council in 1979, almost 45 years ago, to facilitate the coordination and collaboration of MX software developers (Agirre & Dodson, 2018 ▸). Aside from coordinating and distributing software, CCP4 has a mission of promoting the teaching of MX, with an annual didactic CCP4 Study Weekend and numerous online and in-person annual workshops around the world. Forums, which originally took the shape of email lists – the CCP4 bulletin board (or CCP4bb) for general users’ questions and ccp4-dev for developer discussions – are an evolving aspect of the CCP4 community, with social media taking a more prominent role in hosting other kinds of exchanges, for example paper or event announcements (Twitter: @ccp4_mx) or parallel discussions at conferences (Slack channels). The CCP4 website (https://www.ccp4.ac.uk) is the primary mechanism for reference and asynchronous communication but, most importantly, provides a central distribution point for software downloads. A minimal installer package can be obtained from the site, and this will proceed to install the latest version of the suite. Updates are then distributed via a non-disruptive mechanism that was first introduced with *CCP*4 version 6.3.0 in 2012. Update reminders are generated automatically, although the update mechanism itself is, by design, initiated manually. As an indication of update frequency, the 7.0 series, which was originally released in 2016, saw more than 70 updates until the 7.1 series was released in 2020. Updates are not a one-way road: they may be rolled back if problems are encountered. Whilst every effort has been made to keep the suite streamlined and maintainable, the inclusion of large databases and toolkits has driven space requirements steadily upwards (Fig. 1 ▸).

The last decade has seen some large transformations in the field of MX: new workflows have been created (for example phasing with *AlphaFold*2 models) and some old workflows have been optimized, while some others are on the verge of disappearing; this has often been the result of cross-pollination with other techniques in structural biology, for example electron cryo-microscopy (cryo-EM) in particular, through a synergistic collaboration with CCP-EM (Burnley *et al.*, 2017 ▸), the Collaborative Computational Project for Cryo-EM, which repurposes some *CCP*4 code for the cryo-EM community. For example, owing to the deep-learning revolution in computational structure prediction (Jumper *et al.*, 2021 ▸), it is now possible to phase most structures using large predicted fragments or, owing to the accuracy of the method, even to rigid-body fit an initial predicted model into electron density (Oeffner *et al.*, 2022 ▸; McCoy *et al.*, 2022 ▸; Medina *et al.*, 2022 ▸). As a side effect of the creation of these new workflows, experimental phasing is now losing importance in the everyday activities of an MX laboratory, with derivatives only being created as a last resort after all of the now conventional methods have failed. Data acquisition and processing, greatly bolstered by both software and hardware developments *in situ* at synchrotrons, is now performed almost instantaneously after data collection, presenting the user with the results of applying different processing strategies. Although seemingly unconnected, most of these newer developments have one thing in common: the Python programming language as a platform for pipelining and program communication.

While some Python scripts were already part of the *CCP*4 suite even before the time of the last general publication (Winn *et al.*, 2011 ▸), most of the recent source code committed to the CCP4 repositories involves Python in one way or another; for example, both the data-integration tool *DIALS* (Winter *et al.*, 2018 ▸) and its *CCP*4 graphical user interface *DUI* (Fuentes-Montero *et al.*, 2016 ▸) are Python-heavy software. Other *CCP*4 programs, encoded in a different language such as C++ for performance reasons, may also offer Python bindings; examples include *Coot* (Emsley *et al.*, 2010 ▸), *Privateer* (Agirre *et al.*, 2015 ▸) and *GEMMI* (Wojdyr, 2022 ▸), which is a crystallographic toolkit developed in collaboration with Global Phasing Ltd. Both the Python language and its interpreter are now at the core of the *CCP*4 suite. Importantly, both new graphical user interfaces to the *CCP*4 suite (see below) make substantial use of the Python language.

On the subject of graphical user interfaces, a large paradigm shift is also under way, with both *CCP*4*i*2 and *CCP*4 Cloud making extensive use of web technologies: HTML, CSS and JavaScript are used for both interface design and result presentation, with *CCP*4 Cloud making a strong case for the transformation of existing interactive model-building and illustration applications, for example *Coot* and *CCP*4*mg*, into apps that can be run within a web browser.

## 2. Overview of the newest developments

### 2.1. Graphical user interfaces

The long-serving *CCP*4*i* interface (developed in Tcl/Tk) has recently been deprecated and replaced by a more modern, QT/PySide graphical user interface (GUI) named *CCP*4*i*2 (Potterton *et al.*, 2018 ▸). The *CCP*4*i*2 GUI, the main purpose of which is to provide a desktop-based experience, has introduced a number of architectural differences with respect to the first iteration. (i) A real database system, as opposed to a directory structure, provides traceability of files and jobs, and allows the automatic population of inputs to follow-on jobs with outputs from previous jobs. (ii) Large MTZ files are separated into important column sets defining particular data types and with predictable names, for example Miller indices (H, K and L columns) plus amplitudes and estimated standard deviations or e.s.d.s (F and SIGF columns) define an ‘Amplitudes’ data type. (iii) Individual programs are wrapped in Python for their incorporation into tasks, which in many cases will be pipelines themselves; for example ‘Data reduction’ is a pipeline that involves use of the programs *POINTLESS*, *AIMLESS*, *CTRUNCATE* and *FREER*. (iv) Communication of results between individual programs is consolidated in structured data (XML) files. In addition, task reports aim to present only fundamental results and, where possible, provide expert diagnostics in a natural human-readable language, for example ‘No evidence of possible translational noncrystallographic symmetry’. Other utilities include a multiplatform project import and export mechanism, instant job search by keywords, the use of task-specific key performance indicators, for example *R*_work_/*R*_free_, and context-dependent follow-on jobs with automatic selection of input files and default options. Outside the graphical user interface but very much within its infrastructure, the *i*2*run* module provides a command-line mechanism for running *CCP*4*i*2 pipelines, opening the door to batch processing using interface-level decision making.

**Figure 2:**
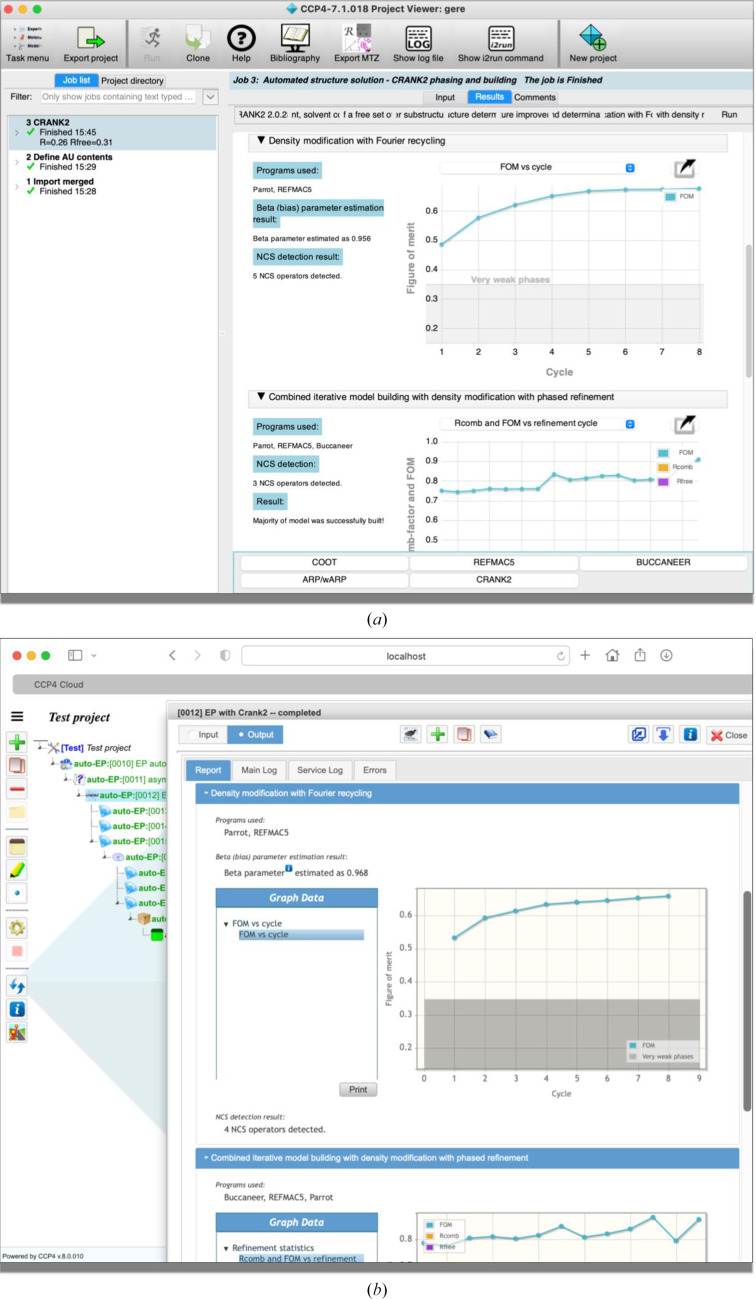
Comparison of the new *CCP*4 graphical user interface offerings: (*a*) desktop (*CCP*4*i*2) and (*b*) online (*CCP*4 Cloud). The same pipeline (*Crank*-2) has been run on both interfaces. The reports show equivalent graphs due to the use of a compatibility layer that allows the same report code to run on both platforms.

*CCP*4 Cloud (Krissinel *et al.*, 2022 ▸) is a complete reimagination of what an interface should look like in the context of macromolecular crystallography. Technology-wise, it provides a server-side JavaScript implementation (based on Node.js) designed to work with high-performance computing (HPC) facilities (clusters and generic clouds) but which can also be run on a user’s PC. This implementation also enables secure web access by a browser via HTML5, CSS and JavaScript (jQuery), and allows *CCP*4 Cloud to look consistent across different browsers and platforms, making it possible to run jobs and manage projects from, for example, mobile devices. The interface provides a general file-import function, which allows it to decide what kind of jobs can be run: for example, automated model building can only be performed if at least reflections and a sequence have been imported. The system features task interfaces for many *CCP*4 programs and some newly introduced pipelines. One such example is *CCP*4*build*, which combines *Parrot* for density modification (Cowtan, 2010 ▸), *Buccaneer* for model building (Cowtan, 2006 ▸), *REFMAC* for refinement (Murshudov *et al.*, 2011 ▸), *Coot* for model editing (Emsley *et al.*, 2010 ▸) and *EDSTATS* (Tickle, 2012 ▸) for model accuracy analysis; using these tools, *CCP*4*build* is able to make expert decisions depending on the phasing approach and model completeness. High-level progress indicators are available in both *CCP*4 Cloud and *CCP*4*i*2; one such example is the ‘verdict’ functionality, which provides a score for model completion and fit to the experimental data. *CCP*4*i*2 and *CCP*4 Cloud have a conceptually similar set of tasks, although their graphical presentation differs (Fig. 2 ▸).

### 2.2. Data processing

Developed in collaboration with Diamond Light Source and the Lawrence Berkeley National Laboratory, the *DIALS* project (Winter *et al.*, 2018 ▸) is the *CCP*4 suite’s main diffraction image processing toolkit; it is modular and hackable by design, so experienced crystallographers can tweak, extend or add new algorithms. Regardless of this specialist component-based approach, complete *DIALS* workflows are provided in the *xia*2 pipeline (Winter, 2010 ▸), which incorporates expert decision making (Winter *et al.*, 2013 ▸). More recently, a graphical user interface (*DIALS User Interface* or *DUI*) has also been introduced (Fuentes-Montero *et al.*, 2016 ▸). The *xia*2 pipeline is run automatically at the end of data collections at Diamond Light Source (Oxfordshire, United Kingdom), providing the results of applying multiple data-processing strategies: users are expected to look at the metrics provided and decide which is better suited to their diffraction data set. Newcomer users wanting to learn more about *DIALS* are advised to use *DUI*, which provides a guided step-by-step execution of the whole process, although command-line use through simple scripts is designed to be accessible to the non-expert user.

*DIALS* is able to natively process data obtained at X-ray free-electron laser (XFEL) facilities (Ginn *et al.*, 2015 ▸; Uervirojnangkoorn *et al.*, 2015 ▸) and supports multi-crystal scaling (Beilsten-Edmands *et al.*, 2020 ▸) and analysis via *xia*2.*multiplex* (Gildea *et al.*, 2022 ▸), serial crystallography (Brewster *et al.*, 2018 ▸; Parkhurst, 2020 ▸) and electron diffraction such as that obtained with standard field emission gun (FEG) cryo-microscopes (Clabbers *et al.*, 2018 ▸). Data from multiple crystals may be scaled and merged together with *BLEND* (Mylona *et al.*, 2017 ▸). Ice rings and further pathologies in measured data can be identified by a separate stand­alone tool named *AUSPEX*, which provides visual and automatic diagnostics based on statistics (Thorn *et al.*, 2017 ▸) and, more recently, machine learning (Nolte *et al.*, 2022 ▸). Alternatively, the *iMosflm* software (Powell *et al.*, 2017 ▸) provides an easy-to-use interface to the *MOSFLM* image-processing program; while the software is no longer under active development, it contains many useful features and remains popular with users.

Once the data have been processed, Laue group determination and data scaling and reduction can be performed directly with *DIALS*, although *POINTLESS* and *AIMLESS* are also offered as a fallback mechanism (Evans & Murshudov, 2013 ▸); indeed, the latter two programs form the basis of the *CCP*4*i*2 ‘data reduction’ task. Further diagnostics can be obtained by running *CTRUNCATE*, which was originally an implementation of French and Wilson’s algorithm (French & Wilson, 1978 ▸), to obtain structure-factor amplitudes from intensities; it will scan data sets for signs of anisotropic diffraction, twinning and translational non­crystallographic symmetry (tNCS) among other critical issues that could complicate or even compromise the downstream structure-determination process. This set of programs has graphical interfaces in both *CCP*4*i*2 and *CCP*4 Cloud, producing colour-coded reports that flag up potential problems. Importantly, detailed reports are generated whenever merged intensities or amplitudes are imported into the graphical interfaces, providing a sanity check and metadata tracking.

### 2.3. Phasing

The *CCP*4 suite provides software for all phasing methods, although they mainly fall within one of the following categories: molecular replacement (MR), *ab initio* phasing with ideal fragments (a special case of molecular replacement) and experimental phasing. In the coming years, and due to the recent improvement in protein structure-prediction methods, the line between the former two is expected to become blurred or even disappear.

#### 2.3.1. Molecular replacement and *ab initio* phasing, including bioinformatics

While the ever-growing area of bioinformatics is outside the remit of *CCP*4, the search for suitable molecular-replacement templates is primarily driven by protein homology analysis and therefore exploits bio­informatics methods. Various third-party tools have been incorporated into the suite to give support to the *CCP*4 model-preparation tools and automated structure-solution pipelines. *MrBUMP* is an automated tool that will perform searches for templates and attempt molecular replacement with them, displaying comprehensive results that can be taken forward provided that the *R* factors are low enough. It can find structures of homologues using *PHMMER* (Eddy, 2011 ▸) or *HHpred* (Söding, 2005 ▸) and place them using either *Phaser* (McCoy *et al.*, 2007 ▸) or *MOLREP* (Vagin & Teplyakov, 2010 ▸). The template search code of *MrBUMP* can also be harnessed interactively in *CCP*4*mg*, allowing users to create composite models and ensembles for subsequent MR searches; this tool can be accessed from both *CCP*4*i*2 and *CCP*4 Cloud. *MrParse* (Simpkin, Thomas *et al.*, 2022 ▸) provides a convenient visual­ization of potential search models from the PDB and databases of new generation models such as the AlphaFold Protein Structure Database (Varadi *et al.*, 2022 ▸). Designed to slice predicted models as well as homologs into domains that may differ in relative orientation from the crystal structure, *Slice’N’Dice* (Simpkin, Elliott *et al.*, 2022 ▸) is an automated molecular-replacement pipeline that facilitates the placement of these domains in molecular replacement. By processing and slicing the models, it simplifies the task of placing these domains. *CCP*4*mg* (McNicholas *et al.*, 2011 ▸) can also be used to visualize the slicing of the input models.

*CCP*4 has a number of efficient molecular-replacement packages: *AMoRe* (Trapani & Navaza, 2008 ▸), *MOLREP* (Vagin & Teplyakov, 2010 ▸) and *Phaser* (McCoy *et al.*, 2007 ▸) all have different strengths, although only the latter is under active development.

*Phaser* uses a maximum-likelihood approach to the phasing problem; it is the only molecular-replacement software that uses intensities natively, *i.e.* without turning them into amplitudes first, and can also use SAD data (for SAD and MR-SAD phasing). The *voyager* (Sammito *et al.*, 2019 ▸) automated procedure within *Phaser* presents a new architecture that allows more flexibility, guiding user decisions in creating ensembles. It also provides, alongside a plethora of new and reimplemented algorithms, code to make the best use of *AlphaFold* (Jumper *et al.*, 2021 ▸) and *RoseTTAFold* (Baek *et al.*, 2021 ▸) structure predictions, or high-confidence subsets of them, including the transformation of model confidence metrics (for example the *AlphaFold* pLDDT) into estimated *B* factors. Owing to the flexibility of the new design, tools for fitting models into cryo-EM maps have been included. An *ad hoc* graphical user interface is under development; this will allow easier navigation of the different solutions calculated during the search strategy, presenting the user with essential plots such as the self-rotation function.

*CCP*4 also has fragment-based *ab initio* phasing packages: *ARCIMBOLDO* (Rodríguez *et al.*, 2009 ▸) and *Fragon* (Jenkins, 2018 ▸), which use ideal fragments of proteins (mainly helices) in targeted molecular-replacement searches. The use of these programs was initially confined to high-resolution data, but they have recently enjoyed success at resolutions lower than 2.3 Å, a threshold beyond which it becomes difficult to ascertain the direction of helical fragments, owing to their improved search strategies (Medina *et al.*, 2022 ▸), phase combination (Millán *et al.*, 2020 ▸) and the use of available structural information, including *AlphaFold* predictions. *ARCIMBOLDO* (Rodríguez *et al.*, 2009 ▸) can use fragments of homologous models and phase previously intractable coiled-coil structures (Caballero *et al.*, 2018 ▸). It should be noted that part of the success of these methods is down to the ability of *Phaser* to place single amino acids or even atoms with great accuracy (McCoy *et al.*, 2017 ▸) and the ability of the density-modification and autotracing algorithms in *SHELXE* (Usón & Sheldrick, 2018 ▸) to bootstrap solutions from poor starting phase sets with average errors as high as 70° (Millán *et al.*, 2015 ▸). Also in alternative MR territory is *AMPLE* (Bibby *et al.*, 2012 ▸), which majors on editing search-model ensembles, particularly *ab initio* predictions and distant homologues.

*SIMBAD* (Simpkin *et al.*, 2018 ▸, 2020 ▸) provides a sequence-independent phasing pipeline that may be used for phasing crystals of unknown contaminants (Simpkin *et al.*, 2018 ▸). Other MR pipelines use larger fragments or domains as their source of phasing information: *BALBES* (Long *et al.*, 2008 ▸) and *MoRDA* (Vagin & Lebedev, 2015 ▸) are automated pipelines that use *MOLREP* to place matches from curated databases containing fragments, domains and homo- and hetero-oligomers. *Dimple* (Wojdyr *et al.*, 2013 ▸) is an automated procedure that aims to quickly arrive at a solved structure of a protein–ligand complex starting from an isomorphous crystal; the software will phase the data and produce preliminary maps, including a difference density map where omit density for a ligand might be found.

#### 2.3.2. Experimental phasing

The steady increase in unique new domains deposited every year in the PDB, the availability of millions of predicted models in the AlphaFold Protein Structure Database (Varadi *et al.*, 2022 ▸) and the continuous improvement of fragment-based *ab initio* phasing methods mean that experimental phasing is increasingly becoming a last-resort approach to recovering phases; it also means that software will have to deal with the most difficult cases. New since the time of the last *CCP*4 general publication (Winn *et al.*, 2011 ▸) is the inclusion of the *SHELXC*/*D*/*E* (Sheldrick, 2008 ▸) programs, which can be run individually or in a pipeline through the *Crank*-2 (Skubák & Pannu, 2013 ▸) frontend, which is available in both the *CCP*4*i*2 and *CCP*4 Cloud interfaces. *Crank*-2 itself incorporates a number of different algorithms that can deal with SAD, SIRAS, MAD and MR-SAD. As stated in the previous section, the *Phaser* software (McCoy *et al.*, 2007 ▸) is also able to perform both SAD and MR-SAD phasing.

### 2.4. Model building and refinement

#### 2.4.1. Interactive model building

The *CCP*4 suite ships with the *de facto* industry-standard interactive model-building program *Coot* (Emsley *et al.*, 2010 ▸). After two decades under constant development, the *Coot* software package has now reached version 1.0, which incorporates a major rework of the graphical architecture, interface, tools and components of the program. Aside from all of the well known tools for manual model building, the software has a built-in ligand building tool *Lidia*, which can use *AceDRG* (see below) for restraint generation, the ability to create covalent linkages between protein and ligand or between molecular components (Nicholls, Joosten *et al.*, 2021 ▸), a semi-automatic *N*-glycan building tool, which is able to build entire oligosaccharides that are consistent with the most common biosynthetic pathways (Emsley & Crispin, 2018 ▸), a real-space, accelerated refinement tool that is able to process whole macromolecules, in contrast to the manual localized real-space refinement that users typically perform when fitting or tweaking parts of a model (Casañal *et al.*, 2020 ▸), and validation tools that run the most common checks on protein models (Ramachandran plots, rotamer propensities, planarity of the peptide bond, per-residue *B* factors and density-fit analysis, amongst others), plus tools to facilitate ligand fitting (Nicholls, 2017 ▸) and validation (Emsley, 2017 ▸), for example deviation from ideal geometry values in dictionaries, clashes and interaction maps. *Coot* makes use of the CCP4 Monomer Library to obtain restraints for the most common biomolecule monomers (amino acids, carbohydrates, nucleic acids) and most ligands defined in the PDB Chemical Component Dictionary (Westbrook *et al.*, 2015 ▸).

At present, *Coot* is tied to desktop machines due to its reliance on the GTK toolkit (Emsley *et al.*, 2010 ▸). This means that users of *CCP*4 Cloud (Krissinel *et al.*, 2022 ▸) need to have a local installation of the *CCP*4 suite in order to perform manual model building. However, there is an ongoing effort to produce a web-based interface, which will use the *Coot* engine in the same manner that the GTK version does but without requiring a local *CCP*4 installation.

#### 2.4.2. Automated model building

While *Coot* has incrementally added a wealth of automatic procedures over the years, the *CCP*4 suite includes several fully automated pipelines that combine automated model-building software [*Buccaneer* (Cowtan, 2006 ▸) and *Nautilus* (Cowtan, 2014 ▸), *ARP*/*wARP* 8.0 (Lamzin *et al.*, 2012 ▸) or the chain-tracing code in *SHELXE* (Usón & Sheldrick, 2018 ▸)] with reciprocal-space refinement (see Section 2.4.4) and validation [*EDSTATS* (Tickle, 2012 ▸) and *MolProbity* (Williams *et al.*, 2018 ▸)] to produce protein and nucleic acid models that are completed iteratively. These pipelines, for example *Modelcraft* (Bond & Cowtan, 2022 ▸) in *CCP*4*i*2 and *CCP*4*build* in *CCP*4 Cloud, are available from both modern graphical user interfaces (*CCP*4*i*2 and *CCP*4 Cloud) and are completed by either graphical or textual summaries of the completeness of the built model. Outside the protein realm, *AlphaFold* (Jumper *et al.*, 2021 ▸) and *RoseTTAfold* (Baek *et al.*, 2021 ▸) models can be glycosyl­ated using the glycan library and tools in the *Privateer* software (Bagdonas *et al.*, 2021 ▸). *PanDDA* (Pearce *et al.*, 2017 ▸) allows users to increase the signal-to-noise ratio of their ligand maps by combining several data sets from ligand-free and ligand-bound forms of the protein; the program has algorithms for combining different crystal forms. The current automated model-building offerings in the suite are completed by *ARP*/*wARP* 8.0 (Lamzin *et al.*, 2012 ▸), which was jointly released with *CCP*4 version 7.0 for the first time in 2018; this software pioneered the iterative combination of model building and refinement (Perrakis *et al.*, 1999 ▸), a feature that is now present in all modern model-building pipelines, and the automated addition of ligands (Langer *et al.*, 2008 ▸). Modern versions of *ARP*/*wARP* may also be used with cryo-EM data (Chojnowski *et al.*, 2021 ▸). At a higher level, the *PDB-REDO* pipeline has been integrated into *CCP*4 through graphical interfaces in *CCP*4*i*2 and *CCP*4 Cloud, with API calls to the *PDB-REDO* web server (Joosten *et al.*, 2014 ▸).

#### 2.4.3. Restraint dictionaries: the CCP4 Monomer Library

The dictionaries in the CCP4 Monomer Library (Vagin *et al.*, 2004 ▸) have been improved by the introduction of *AceDRG* (Long *et al.*, 2017 ▸), which since version 7.0 of the suite can also generate restraint dictionaries for covalent linkages (Nicholls, Wojdyr *et al.*, 2021 ▸; Nicholls, Joosten *et al.*, 2021 ▸). New dictionaries are now routinely generated for many compounds, although pyranose sugars have received a separate treatment to account for their conformational preferences (Atanasova *et al.*, 2022 ▸; Joosten *et al.*, 2022 ▸). H atoms have been modelled and restrained in their nuclear positions in the CCP4 Monomer Library (Catapano *et al.*, 2021 ▸), as informed by neutron diffraction data (Allen & Bruno, 2010 ▸).

#### 2.4.4. Refinement

The main tool for full-model reciprocal-space refinement in *CCP*4 is *REFMAC*5 (Murshudov *et al.*, 2011 ▸). The program uses the sparse-matrix approximation of the Fisher’s information matrix (Steiner *et al.*, 2003 ▸) and is designed to be fast and flexible, with a number of refinement methods built into the engine, including restrained, un­restrained and rigid-body refinement. Jelly-body restraints are particularly useful for stabilizing refinement, for example, after molecular replacement, where larger parts of a structure might need to move into place. In addition to controlling model parameterization and performing macromolecular refinement, *REFMAC*5 also performs map calculation. A variety of types of weighted maps are produced, which allow visualization, subsequent analyses and validation.

*REFMAC*5 allows the addition of case-specific structural knowledge to be utilized during refinement through the external restraints mechanism (Nicholls *et al.*, 2012 ▸; Kovalevskiy *et al.*, 2018 ▸). These external restraints, which are most useful when only low-resolution data are available, can for instance be generated by *ProSMART* (Nicholls *et al.*, 2014 ▸) for proteins and nucleic acids using homologues or backbone hydrogen-bonding patterns, *LibG* (Brown *et al.*, 2015 ▸) for nucleic acid base-pairing and stacking, and *Platonyzer* (Touw *et al.*, 2016 ▸) for zinc, sodium and magnesium sites. The automated pipeline *LORESTR* (Kovalevskiy *et al.*, 2016 ▸) can be used to optimize the refinement protocol at low resolution, expediting the process and easing manual user effort. New developments and the next generation of structure-refinement tools are being implemented in *Servalcat* utilizing the *GEMMI* library (Yamashita *et al.*, 2021 ▸, 2023 ▸).

The *PAIREF* program (Malý *et al.*, 2020 ▸), which has recently been introduced into *CCP*4*i*2, performs automatic paired refinement (Karplus & Diederichs, 2012 ▸) using the *REFMAC*5 refinement engine. It analyses the impact of weak reflections beyond the traditional high-resolution diffraction-limit cutoff on the quality of the refined model. The program monitors model and data indicators and model-to-data agreement metrics and implements a decision-suggesting routine for the high-resolution cutoff that may result in the best model. Outside *REFMAC*5 and associated tools, the *SHEETBEND* software (Cowtan *et al.*, 2020 ▸) allows a very fast preliminary refinement of the atomic coordinates and, optionally, isotropic or anisotropic *B* factors (Cowtan & Agirre, 2018 ▸). It is based on a novel approach in which a shift field, and not atoms, is refined to update and morph models. This approach is particularly indicated to correct large shifts in secondary-structure elements after molecular replacement and is run by default as part of the *Modelcraft* pipeline (Bond & Cowtan, 2022 ▸).

### 2.5. Validation and deposition

Both the *CCP*4*i*2 and *CCP*4 Cloud interfaces include a validation and deposition interface developed in collaboration with the PDBe (the Protein Data Bank in Europe; wwPDB Consortium, 2019 ▸; Armstrong *et al.*, 2020 ▸). The purpose of this tool is to prepare mmCIF files for deposition; additionally, it provides the convenience of letting users see what their preliminary wwPDB validation report (Gore *et al.*, 2012 ▸, 2017 ▸) would look like and allowing them to fix errors and notice interesting chemical features of a model before going through the actual deposition process. Also, in preparation for deposition, the model and structure factors are converted into an mmCIF, which in turn allows the wwPDB to pre-populate many of the required metadata for deposition, such as refinement statistics.

Further validation tools exist in *CCP*4 outside this online validation process. Protein model validation can be performed with a variety of tools. *MolProbity* analyses backbone geometry, rotamers and clashes, and produces a script file that will generate a menu within *Coot* containing lists of outliers. *Coot* itself contains a plethora of interactive and live-updated validation tools, ranging from *MolProbity*-equivalent metrics to other less frequently quoted metrics, for example the Kleywegt Plot, which can be of great value depending on the problem. The *EDSTATS* software (Tickle, 2012 ▸) provides a unique analysis of model-to-data fit, separating results by main chain and side chain and looking at difference density, with the results being able to point out common modelling problems, such as poorly fitting regions requiring a peptide flip. Version 8.0 of *CCP*4 has seen the gradual inclusion of *PDB-REDO* (Joosten *et al.*, 2012 ▸) functionality into the *CCP*4 interfaces; for example *Tortoize* (Sobolev *et al.*, 2020 ▸), a tool that analyses main-chain and side-chain geometry and reports *Z*-scores for every amino acid, is now integrated into the *CCP*4 validation tasks. The visual output of *PDB-REDO* calculations is displayed consistently across *CCP*4*i*2, *CCP*4-Cloud and the *PDB-REDO* website by encapsulating various interactive plots and tables in a self-contained single web component. Detection of errors, particularly sequence-register errors, by analysing the agreement between observed contacts and inter-residue distances with the predictions from software such as *AlphaFold*2 (Sánchez Rodríguez *et al.*, 2022 ▸) is available in *ConKit* (Simkovic *et al.*, 2017 ▸). The *findMySequence* software (Chojnowski *et al.*, 2022 ▸) uses machine learning for the identification of unknown proteins in X-ray crystallography and cryo-EM data, with the added benefit of detecting elusive register errors, which may have a detrimental effect on the quality of the rest of the structure. The *Iris* validation framework (Rochira & Agirre, 2021 ▸) is a standalone tool that displays a variety of validation metrics as concentric circles, with modelling errors becoming visible as ripples in successive circles. Carbohydrate model validation, including protein glycosylation, can be carried out with the *Privateer* software (Agirre *et al.*, 2015 ▸), which in the MKIV version incorporates checks of glycan composition against offline mirrors of several glycomics databases (Bagdonas *et al.*, 2020 ▸) and overall glycan conformation using *Z*-scores (Dialpuri *et al.*, 2023 ▸). Specific structural radiation-damage sites in structures derived from cryocooled crystals can be identified with *RABDAM* through the *B*_damage_ (Shelley *et al.*, 2018 ▸) and *B*_net_ (Shelley & Garman, 2022 ▸) metrics, and space-group and origin ambiguity may be determined and resolved using *Zanuda* (Lebedev & Isupov, 2014 ▸).

### 2.6. Analysis and representation

*PISA* (Krissinel & Henrick, 2007 ▸) allows the analysis of molecular interfaces, calculating likely assemblies, intra­molecular and intermolecular contacts, and accessible areas, offering insight into crystal packing. Intramolecular (predicted) contact maps and other related representations can be visualized with *ConKit* (Simkovic *et al.*, 2017 ▸) or online at the *ConPlot* server (Sánchez Rodríguez *et al.*, 2021 ▸).

**Figure 3:**
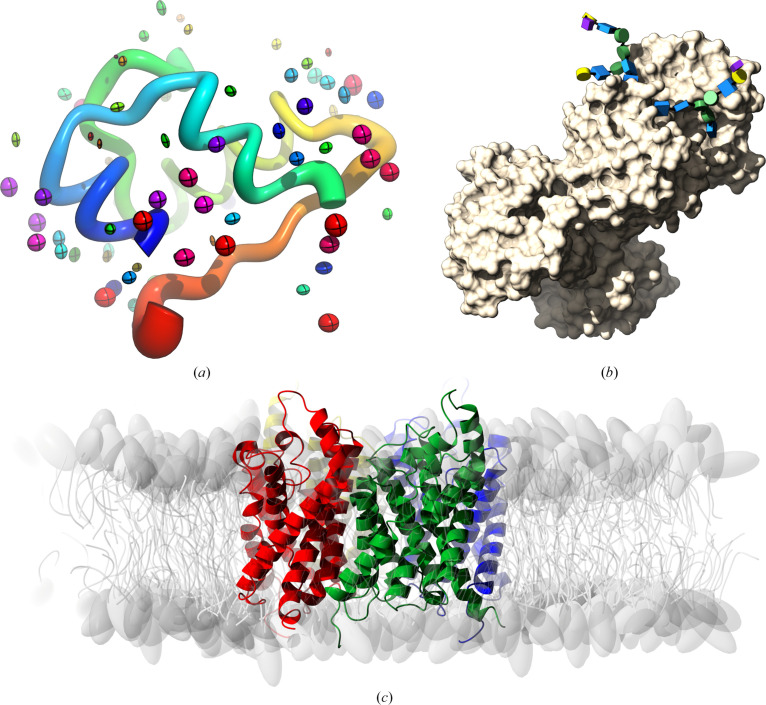
A collection of newer representations included in the *CCP*4 *Molecular Graphics* project (*CCP*4*mg*). (*a*) PDB entry 2bn3 is a high-resolution model of insulin (Nanao *et al.*, 2005 ▸); it is shown here as worms, with water molecules drawn as ellipsoids, both coloured and scaled by the anisotropic *B* factors of the model. (*b*) PDB entry 3v8x (Noinaj *et al.*, 2012 ▸) is a structure of human transferrin (chain *B*), drawn here as a solvent-accessible surface with *N*-glycans shown as Glycoblocks (McNicholas & Agirre, 2017 ▸). (*c*) PDB entry 3c02, a structure of aquaglyceroporin from *Plasmodium falciparum* (Newby *et al.*, 2008 ▸), embedded in a lipid bilayer by *CHARMM-GUI* (Jo *et al.*, 2008 ▸); lipids are shown as cartoons.

On the representation side, the main tool in *CCP*4 is the *CCP*4 *Molecular Graphics* project (*CCP*4*mg*). Since the last *CCP*4*mg* general publication (McNicholas *et al.*, 2011 ▸), the main updates have involved new functionalities for handling cryo-EM maps, 3D representation of *N*-glycans (McNicholas & Agirre, 2017 ▸) and the addition of a new interactive interface to the functionality of *MrBUMP* (Keegan *et al.*, 2018 ▸). Some newer representations from *CCP*4*mg* can be seen in Fig. 3 ▸.

### 2.7. Under the bonnet

The *dxtbx* toolkit for *DIALS* (Parkhurst *et al.*, 2014 ▸) is included as part of the *cctbx* (Grosse-Kunstleve *et al.*, 2002 ▸) distribution; the *clipper-python* module (McNicholas *et al.*, 2018 ▸), a SWIG wrapper around the original C++ Clipper library, is also included and supports a number of functions of the *CCP*4*i*2 interface, including the *Iris* validation framework (Rochira & Agirre, 2021 ▸). At a higher level, *CCP*4*i*2 (Potterton *et al.*, 2018 ▸) provides code reusability via the command line, offering a mechanism for executing Python-only pipelines without a running instance of the graphical user interface (headless mode). *CCP*4 Cloud projects and automatic structure-solution workflows can also be initiated from the command line using the ‘cloudrun’ utility; this is useful for performing serial computations for selected targets. The *Coot* model-building software (Emsley & Cowtan, 2004 ▸), originally conceived as a C++ object-oriented toolkit, is now exposed as an importable Python module to allow code reuse in new applications, and is also able to run in headless mode, suppressing all graphical output. Finally, *CCP*4*mg* (Mc­Nicholas *et al.*, 2011 ▸) is also able to run without graphics, generating images from a scene-description file in XML format; this functionality is used in *CCP*4*i*2 to generate molecular graphics of, for instance, autobuilt structures.

## 3. Future plans

The transition towards web technologies, which is already under way with the introduction of *CCP*4 Cloud, will be completed in the near future by the introduction of fully fledged model-building, visualization and figure-preparation web-browser interfaces to the existing *Coot* and *CCP*4*mg* engines. We also foresee an increase in the number of connections to theoretical modelling packages such as *AlphaFold* (Jumper *et al.*, 2021 ▸) and *RoseTTAfold* (Baek *et al.*, 2021 ▸), as well as deeper harnessing of the AlphaFold Protein Structure Database (Varadi *et al.*, 2022 ▸).

## 4. Software availability and data-access statement

The *CCP*4 software suite can be obtained from https://www.ccp4.ac.uk/download. CCP4 maintains a public instance of *CCP*4 Cloud at https://cloud.ccp4.ac.uk available to both academic and licenced commercial users. No data were generated in the context of the present publication.

## 5. Individual author contributions

Jon Agirre wrote the majority of the manuscript, coordinated the authors and contributed to *Privateer*, *clipper-python*, *clipper-progs*, *CCP*4*i*2, *CCP*4 Cloud, *Iris*, the CCP4 Monomer Library and other software. Haroldas Bagdonas contributed to *Privateer* MKIV. James Beilsten-Edmands, Luis Fuentes-Montero, Markus Gerstel, Richard J. Gildea, James M. Parkhurst, Nicholas E. Devenish, Melanie Vollmar, David Waterman, Graeme Winter and Gwyndaf Evans contributed to *xia*2 (Winter) and *DIALS*. James Foadi and Gwyndaf Evans developed *BLEND*. Rafael J. Borges, Claudia Millán, Iracema Caballero, Elisabet Jiménez, Josep Triviño Valls and Isabel Usón developed the *ARCIMBOLDO* package, with Massimo Sammito and Ana Medina contributing to *ALEPH*. George Sheldrick is the lead developer of *SHELXC*/*D*/*E*; Isabel Usón is now the main contributor to and maintainer of the *SHELXC*/*D*/*E* suite. Maarten L. Hekkelman, Robbie P. Joosten and Anastassis Perrakis developed the *PDB-REDO* software package. Paul Bond, Soon Wen Hoh and Kevin D. Cowtan contributed to *Modelcraft* and *Buccaneer* (Bond, Hoh and Cowtan), *Nautilus* (Hoh and Cowtan) and the Clipper libraries (Cowtan). Tristan I. Croll, Soon Wen Hoh, Stuart McNicholas and Jon Agirre led the development of the released *clipper-python* module. J. Javier Burgos-Mármol, Ronan M. Keegan, Filomeno Sánchez Rodríguez, Felix Simkovic, Adam J. Simpkin, Jens M. H. Thomas and Daniel J. Rigden developed *SIMBAD*, *MrBUMP*, *ConKit*, *Slice’N’Dice* and *AMPLE*. Stuart J. McNicholas, Kyle Stevenson, Huw T. Jenkins, Eleanor J. Dodson, Keith S. Wilson and Martin E. M. Noble contributed to the development and testing of the *CCP*4*i*2 graphical user interface. John Berrisford and Sameer Velankar contributed towards the development of a validation and deposition task in the *CCP*4 graphical user interfaces. Paul Emsley is the lead developer of *Coot* and associated programs, to which Bernhard Lohkamp has contributed. William Rochira developed *Iris* under Jon Agirre’s supervision. Nicholas Pearce contributed *PanDDA* to the suite. Philipp Heuser, Joana Pereira, Egor Sobolev, Grzegorz Chojnowski and Victor S. Lamzin contributed to *ARP*/*wARP* 8.0. Pavol Skubak and Navraj S. Pannu developed *Crank*-2. Oleg Kovalevskiy is the lead developer of *LORESTR*. Fei Long is the lead developer of *AceDRG*, *BALBES* and *LibG*. Garib N. Murshudov is the lead developer of *REFMAC*5. Robert A. Nicholls is the lead developer of *ProSMART*. Mihaela Atanasova, Lucrezia Catapano, Robbie P. Joosten, Andrey A. Lebedev, Fei Long, Stuart J. McNicholas, Garib N. Murshudov, Robert A. Nicholls, Roberto A. Steiner and Keitaro Yamashita contributed to *REFMAC*5 and/or the CCP4 Monomer Library. Andrew G. W. Leslie and Harold R. (Harry) Powell led the development of *MOSFLM* and *iMosflm*, respectively. Andrea Thorn is the lead developer of *AUSPEX*. Phil R. Evans is the developer of *POINTLESS* and *AIMLESS*. Alexei Vagin was the lead developer of *MoRDA*. Airlie J. McCoy, Kaushik Hatti, Robert Oeffner, Massimo Sammito, Claudia Millán and Randy J. Read developed *Phaser* and the associated tools. Eugene Krissinel developed *PISA*, *SSM*, *Gesamt* and, with Andrey A. Lebedev and others, the *CCP*4 Cloud software. Martin Malý and Petr Kolenko designed and implemented the *PAIREF* software. Kathryn L. Shelley and Elspeth F. Garman led the development of *RABDAM*. Maria Fando developed a new documentation architecture for *CCP*4*i*2 and *CCP*4 Cloud and converted, with help from others, old documentation to the new system. Gregorz Chojnowski developed the *findMySequence* software. Martyn Winn wrote the original implementation of TLS refinement in *REFMAC* and contributed to the development of the core C libraries and to *MrBUMP*.

At the time of writing, the CCP4 Executive Committee was composed of David G. Brown, Helen Walden, Kevin D. Cowtan, Judit Debreczeni, Gwyndaf Evans, Michael A. Hough, Dave Lawson, James Murray, Martyn D. Winn, Garib N. Murshudov, Martin E. M. Noble, Randy J. Read, Dan J. Rigden, Ivo Tews, Eugene Krissinel and Keith S. Wilson. Jon Agirre and Arnaud Baslé were subsequently elected as co-chairs of CCP4 Working Group 2 and took seats on the CCP4 Executive Committee, of which Ivo Tews was elected as chair. Charles B. Ballard, Ronan M. Keegan, Andrey A. Lebedev, Maria Fando, Tarik R. Drevon, David Waterman, Ville Uski and Eugene B. Krissinel were the members of the CCP4 Core Team responsible for the maintenance and distribution of the *CCP*4 software suite, *CCP*4 Cloud and website.
